# Mitochondrial genome nucleotide substitution pattern between domesticated silkmoth, *Bombyx mori*, and its wild ancestors, Chinese *Bombyx mandarina* and Japanese *Bombyx mandarina*

**DOI:** 10.1590/S1415-47572009005000108

**Published:** 2010-03-01

**Authors:** Yu-Ping Li, Wu Song, Sheng-Lin Shi, Yan-Qun Liu, Min-Hui Pan, Fang-Yin Dai, Cheng Lu, Zhong-Huai Xiang

**Affiliations:** 1 College of Bioscience and Biotechnology, Shenyang Agricultural University, Liaoning, ShenyangChina; 2 The Key Sericultural Laboratory of Agricultural Ministry, Southwest University, ChongqingChina

**Keywords:** nucleotide substitution pattern, mitochondrial genome, *Bombyx mori*, *Chinese Bombyx mandarina*, Japanese *Bombyx mandarina*

## Abstract

*Bombyx mori* and *Bombyx mandarina* are morphologically and physiologically similar. In this study, we compared the nucleotide variations in the complete mitochondrial (mt) genomes between the domesticated silkmoth, *B. mori*, and its wild ancestors, Chinese *B. mandarina* (Ch*Bm*) and Japanese *B. mandarina* (Ja*Bm*). The sequence divergence and transition mutation ratio between *B. mori* and Ch*Bm* are significantly smaller than those observed between *B. mori* and Ja*Bm*. The preference of transition by DNA strands between *B. mori* and Ch*Bm* is consistent with that between *B. mori* and Ja*Bm*, however, the regional variation in nucleotide substitution rate shows a different feature. These results suggest that the Ch*Bm* mt genome is not undergoing the same evolutionary process as Ja*Bm*, providing evidence for selection on mtDNA. Moreover, investigation of the nucleotide sequence divergence in the A+T-rich region of *Bombyx* mt genomes also provides evidence for the assumption that the A+T-rich region might not be the fastest evolving region of the mtDNA of insects.

The mitochondrial (mt) DNA of insects is a self-replicating, circular DNA molecule about 14-20 kb in size, encoding a conserved set of 37 genes (13 protein genes, 22 tRNA genes, and two rRNA genes) and an A+T-rich segment known as control region (Wolstenholme, 1992; Boore, 1999). Several comparative mt genomics studies for investigation of the nucleotide variation and evolutionary patterns have been carried out, including *Drosophila melanogaster* subgroup members (Ballard, 2000), *Ostrinia nubilalis* and *O. furnicalis* (Coates *et al.*, 2005), as well as *Bombyx mori* and its close relative Japanese *B. mandarina* (Yukuhiro *et al.*, 2002). Generally, the A+T-rich region shows a higher level of sequence variability than other regions of the genome (Fauron and Wolstenholme, 1980; Shao *et al.*, 2001)

The mulberry silkworm *Bombyx mori* is the only truly domesticated insect. It is thought to be domesticated from the wild mulberry silkworm, *B. mandarina*, about 5,000-10,000 years ago (Goldsmith *et al.*, 2005). *B. mori* and *B. mandarina* are morphologically and physiologically similar. However, two types of wild mulberry silkworms with uniform morphology and different chromosome numbers per haploid genome are currently found in mulberry fields. The Japanese *B. mandarina* (Ja*Bm*) living in Japan and Korea has 27 chromosomes in its haploid genome, whereas the Chinese *B. mandarina* (Ch*Bm*), distributed over China, has 28 chromosomes per haploid genome, like its domesticated counterpart *B. mori* that also carries 28 chromosomes (Astaurov *et al.*, 1959). Ja*Bm* was thought to be a probable wild ancestor of *B. mori*, and two sets of biased patterns in the mt genome nucleotide substitution between the two have been confirmed, *i.e.*, a difference in preference of transitional changes between major and minor DNA strands and a variation in the degree of nucleotide substitution by genomic regions (Yukuhiro *et al.*, 2002).

Phylogenetic analysis (Arunkumar *et al.*, 2006; Pan *et al.*, 2008) and historical records (Goldsmith *et al.*, 2005) make it clear that the domesticated silkworm was directly domesticated from the Chinese wild silkworm rather than from the Japanese wild silkworm. We determined the complete mt genome of Ch*Bm* (GenBank accession number: AY301620) that contains a typical gene complement, order, and arrangement identical to that of *B. mori* and Ja*Bm* (Pan *et al.*, 2008). In the present study, we examined the nucleotide variation pattern of the complete mt genome between *B. mori* and Ch*Bm*. The purpose of this study was to determine whether the nucleotide variation pattern between *B. mori* and its wild ancestor, Ch*Bm*, is consistent with that between *B. mori* and its close relative, Ja*Bm*.

The mt genome sequences of *B. mori* C108 (AB070264), Backokjam (NC_002355), Aojuku (AB083339), Xiafang (AY048187), and Ja*Bm* (AB070263) were downloaded from GenBank, aligned by Clustal X (Thompson *et al.*, 1997), and the alignment was analyzed by MEGA version 4.1 (Tamura *et al.*, 2007), including the variable nucleotide sites and the substitution sites.

The four mt genome sequences of *B. mori* C108 (15,656 bp), Backokjam (15,643 bp), Aojuku (15,635 bp), and Xiafang (15,664 bp) were aligned to produce a 15,685 nt consensus alignment, of which only 89 (0.57%) nucleotide sites are variable, including insertions-deletions (indels) (data not shown). Within the four *B. mori* strains, only 33 substitutions were detected, including 24 transition (ts) and 9 transversion (tv) mutations. The variable nucleotide sites ranged from 63 to 74 nt when each two of the four *B. mori* mt genome sequences were compared, showing no significant differences in variable nucleotide rate (χ^2^ = 1.25, *d.f.* = 5, p > 0.05).

However, the mt genome sequences of Ch*Bm* (15,682 bp) and Ja*Bm* (15,928 bp) were aligned to produce a 15,970 nt consensus alignment, of which 639 (4.00%) nucleotide sites are variable including indels, and 325 substitutions were detected. Compared to these data observed within the four *B. mori* strains, the sequence divergence within *B. mandarina* is significantly greater (4.00% *vs* 0.57%; χ^2^ = 415.26, *d.f.* = 1, p < 0.001). Moreover, we identified 484 variable nucleotide sites including indels (3.08% sequence divergence) from a 15,722 nt consensus alignment between the mt genomes of *B. mori* C108 and Ch*Bm*, whereas 855 variable nucleotide sites including indels (5.35% sequence divergence) were identified from a 15,970 nt consensus alignment between *B. mori* C108 and Ja*Bm*. Compared to the sequence divergence observed between *B. mori* C108 and Ja*Bm*, that between *B. mori* and Ch*Bm* was significantly smaller (3.08% *vs* 5.35%; χ^2^ = 101.36, *d.f.* = 1, p < 0.001). These results led us to further compare the nucleotide substitution pattern between *B. mori* C108 and Ch*Bm* with that between *B. mori* C108 and Ja*Bm*.

A total of 381 substitutions were detected between the mt genomes of *B. mori* C108 and Ch*Bm*: 230 transition and 151 transversion mutations (ts:tv = κ ≈ 230/151 = 1.52). This value is significantly deviatory from neutral expectation (1:2, χ^2^ = 125.30, *d.f.* = 1, p < 0.001), indicating that evolutionary pressures are acting upon the two mt genomes. However, between *B. mori* C108 and Ja*Bm*, 514 substitutions were detected: 414 transition and 100 transversion mutations (κ ≈ 4.14), also significantly deviated from neutral expectation (1:2, χ^2^ = 517.47, *d.f.* = 1, p < 0.001). Excess transition mutation was also reported in *Drosophila* (κ = 761/180 ≈ 4.23) and *Ostrinia* (κ = 134/48 ≈ 2.88) mt genomes, and attributed to non-neutral evolutionary forces or population effects (Ballard, 2000; Coates *et al.*, 2005). Compared to the data observed between *B. mori* and Ja*Bm*, the transition mutation ratio between *B. mori* and Ch*Bm* was significantly smaller (1.52 *vs* 4.14) (χ^2^ = 44.14, *d.f.* = 1, p < 0.001), suggesting that their nucleotide substitution pattern is different.

The nucleotide differences between *B. mori* and Ch*Bm* mt genes except for tRNA genes are shown in Table 1. The numbers and rates of nucleotide difference between the mt genes of *B. mori* and Ch*Bm* were found to be decreased compared to those seen between *B. mori* and Ja*Bm*, except for the *srRNA* gene and A+T-rich region, in which the numbers and rates of nucleotide difference were increased. The full *srRNA* sequences of *B. mori* C108 and Ch*Bm* were aligned to produce a 785 nt consensus alignment, of which 12 substitutions were detected, showing a higher nucleotide sequence divergence than the *lrRNA* gene. Comparing *B. mori* C108 and Ch*Bm*, the NADH dehydrogenase subunit encoding *nad*6 gene was the most conserved among genes or regions, followed by the ATP synthase F0 subunit encoding *atp*8 gene. The cytochrome b (*cob*) gene was the most variable, followed by the cytochrome *c* oxidase subunit encoding *cox*3 gene. The nucleotide sequence divergence in the A+T-rich region was moderate among these genes or regions. However, comparing the mt genes of *B. mori* and Ja*Bm,* the *srRNA* gene was the most conserved among genes or regions, and nucleotide sequence divergences in the *lrRNA* gene and the A+T-rich region were also low, compared to those of protein-coding genes.

In this study, we found no significant differences in transition rates for the protein-coding genes encoded by the major strand (22 A-G and 101 C-T transitions in 6,909 bp) between the mt genomes of *B. mori* C108 and Ch*Bm*, compared to those encoded by the minor strand (48 A-G and 13 C-T transitions in 4296 bp) (χ^2^ = 2.13, *d.f.* = 1, p > 0.05). However, on the major strand there were significantly more changes for T-C transitions in protein-coding genes than observed on the minor strand (χ^2^ = 35.35, *d.f.* = 1, p < 0.001), while on the minor strand there were significantly more changes for A-G transitions than observed on the major strand (χ^2^ = 27.23, *d.f.* = 1, p < 0.001). These data are consistent with those previously reported (Ballard 2000; Yukuhiro *et al.*, 2002).

The rates of nucleotide substitutions between the mt genomes of *B. mori* C108 and Ja*Bm* vary by genomic region: the five genes surrounding the A+T-rich region and two rRNA genes (Class 1: *nad*2*, cox*1*, cox*2*, atp*6*,* and *nad*1) are more conserved than the remaining genes (Class 2: *cox*3*, nad*4*, nad*5*,* and *cob*), whereas no significant difference in substitution rates within Classes 1 and Classes 2 has been detected (Yukuhiro *et al.*, 2002). In this study, we found that, between the mt genomes of *B. mori* and Ch*Bm*, there is a significant difference in the nucleotide substitution rate for the 13 protein-coding genes (χ^2^ = 39.68, *d.f.* = 12, p < 0.005), which is consistent with that found between *B. mori* and Ja*Bm*. Further analysis showed that between the mt genomes of *B. mori* and Ch*Bm* the average substitution rate of Class 1 (0.0195) was significantly smaller than that of Class 2 (0.0308) (χ^2^ = 12.70, *d.f.* = 1, p < 0.005); moreover, a significant difference in the substitution rate within Classes 2 was detected (χ^2^ = 15.30, *d.f.* = 3, p < 0.005), but there was no significant difference in substitution rates within Classes 1 (χ^2^ = 0.77, *d.f.* = 4, p > 0.9).

The A+T-rich region is the only major non-coding region in the mt genomes of animals. Based on the assumption that the A+T-rich region is the fastest evolving region of the mtDNA (Zhang and Hewitt, 1997; Giuffra *et al.*, 2000), it has become popular as a molecular marker for population genetic and phylogeographic studies of animals, including insects. However, according to Zhang and Hewitt (1997), in terms of nucleotide substitution, the A+T-rich region might not be the fastest evolving region of the mtDNA of insects. Between the mt genes of *B. mori* and Ja*Bm*, the nucleotide sequence divergence in the A+T-rich region is low, higher only than that of the *srRNA* gene, whereas between *B. mori* and Ch*Bm*, the A+T-rich region presents a moderate sequence divergence, lower only than those of the three protein-coding genes *cob*, *cox*3, and *nad*4L. These observations of nucleotide sequence divergence in the A+T-rich region showed that this is not the fastest evolving gene or region in *Bombyx* mt genomes, providing direct evidence for the above mentioned assumption of Zhang and Hewitt (1997).

The primary finding of this study was that the nucleotide substitution pattern between the mt genomes of *B. mori* and Ch*Bm* is different from that between *B. mori* and Ja*Bm*. Although the preference of transition by DNA strands between *B. mori* and Ch*Bm* was consistent with that between *B. mori* and Ja*Bm*, the regional variation in nucleotide substitution rate showed a different feature. Comparative mt genomics revealed a lower level of transition mutation (ts:tv = 1.52) between *B. mori* and Ch*Bm*, but a significantly higher level of transition mutation (ts:tv = 4.14) between *B. mori* and Ja*Bm*. It has been suggested that the A+T richness in the mt genome would cause an apparently lower transition bias ratio in closely related species (Tamura, 1992; Yu *et al.*, 1999; Liao and Lu, 2000; Arunkumar *et al.*, 2006). However, the present investigation of the A+T content of the mt genome from the three samples showed that they were almost identical (from 81.36% in *B. mori* to 81.68% in Ch*Bm*), indicating that the heavy bias towards A+T is not suitable to explain this case. Therefore, the distinct nucleotide substitution pattern suggests that the mt genomes of Ch*Bm* and Ja*Bm* are the result of different evolutionary forces. The mechanism responsible for that difference remains unclear. A possible explanation is that the environmental selection effect could lead to different mutational biases. In line with previous reports (Messier and Stewart, 1997; Wise *et al.*, 1998; Creevey and McInerney, 2002; Jansa *et al.*, 2003), this study presents evidence for mtDNA selection.

## Figures and Tables

**Table 1:** Nucleotide differences between mitochondrial genes of *B. mori* and Chinese *B. mandarina*, except for tRNA genes.

Genes or regions	Base pairs	Transitional changes				Transversional changes					Sum I	Sum II
		A-G	T-C	Total		T-A	C-A	T-G	C-G	Total		
*nad*2	1,023	2	13	15		2	3	0	0	5	20 (0.0196)	32 (0.0313)
*cox*1	1,535	7	15	22		4	4	2	1	11	33 (0.0215)	46 (0.0300)
*cox*2	682	0	9	9		3	2	0	0	5	14 (0.0205)	21 (0.0308)
*atp*8	162	0	2	2		0	0	0	0	0	2 (0.0123)	8 (0.0494)
*atp* 6	678	0	6	6		3	2	1	0	6	12 (0.0177)	21 (0.0310)
*cox*3	789	3	20	23		7	2	1	0	10	33 (0.0418)	43 (0.0545)
*nad*3	351	1	6	7		1	1	0	0	2	9 (0.0256)	10 (0.0285)
*nad*6	531	0	4	4		1	0	0	0	1	5 (0.0094)	12 (0.0226)
*cob*	1,152 (1,158)	9	26	35		7	3	4	1	15	50 (0.0434)	55 (0.0477)
*nad*5	1,719	25	4	29		5	0	4	1	10	39 (0.0227)	71 (0.0413)
*nad*4	1,341	13	7	20		7	0	4	1	12	32 (0.0238)	51 (0.0380)
*nad*4L	291	3	0	3		3	0	3	0	6	9 (0.0309)	12 (0.0412)
*nad*1	945	7	2	9		1	0	3	3	7	16 (0.0169)	24 (0.0257)
*lrRNA*	1,378 (1,350)	12	1	13		4	0	1	0	5	18 (0.0133)	26 (0.0189)
*srRNA*	783 (784)	3	1	4		7	1	0	0	8	12 (0.0153)	7 (0.0089)
A+T-rich region	494 (484)	7	0	7		5	1	0	0	6	13 (0.0269)	8 (0.0162)

Note: The numbers in parentheses in the second column indicate the gene size of Chinese *B. mandarina*, when gene sizes vary between Chinese *B. mandarina* and *B. mori* C108. Protein coding genes *nad*2, *cox*1, *cox*2, *atp*8, *atp* 6, *cox*3, *nad*3, *nad*6 and *cob* are encoded by the major strand, whereas *nad*5, *nad*4, *nad*4L and *nad*1are encoded by the minor strand. Genes *lrRNA* and *srRNA* are encoded by the minor strand. Data in the Sum I column resulted from the comparison between mitochondrial genes of *B. mori* C108 and Chinese *B. mandarina*; data in the Sum II column resulted from the comparison between *B. mori* C108 and Japanese *B. mandarina* (Yukuhiro *et al.*, 2002).
